# B Quiet: Autoantigen-Specific Strategies to Silence Raucous B Lymphocytes and Halt Cross-Talk with T Cells in Type 1 Diabetes

**DOI:** 10.3390/biomedicines9010042

**Published:** 2021-01-06

**Authors:** Jamie L. Felton, Holly Conway, Rachel H. Bonami

**Affiliations:** 1 Department of Pediatrics, Division of Pediatric Endocrinology and the Herman B. Wells Center for Pediatric Research, Indianapolis, IN 46202, USA; jamifelt@iu.edu (J.L.F.); haconway@iu.edu (H.C.); 2 Department of Medicine, Division of Rheumatology and Immunology, Vanderbilt University Medical Center, Nashville, TN 37232, USA; 3 Department of Pathology, Microbiology and Immunology, Vanderbilt University Medical Center, Nashville, TN 37232, USA

**Keywords:** antibodies, autoantigen, autoimmunity, B cells, insulin, lymphocytes, type 1 diabetes

## Abstract

Islet autoantibodies are the primary biomarkers used to predict type 1 diabetes (T1D) disease risk. They signal immune tolerance breach by islet autoantigen-specific B lymphocytes. T-B lymphocyte interactions that lead to expansion of pathogenic T cells underlie T1D development. Promising strategies to broadly prevent this T-B crosstalk include T cell elimination (anti-CD3, teplizumab), B cell elimination (anti-CD20, rituximab), and disruption of T cell costimulation/activation (CTLA-4/Fc fusion, abatacept). However, global disruption or depletion of immune cell subsets is associated with significant risk, particularly in children. Therefore, antigen-specific therapy is an area of active investigation for T1D prevention. We provide an overview of strategies to eliminate antigen-specific B lymphocytes as a means to limit pathogenic T cell expansion to prevent beta cell attack in T1D. Such approaches could be used to prevent T1D in at-risk individuals. Patients with established T1D would also benefit from such targeted therapies if endogenous beta cell function can be recovered or islet transplant becomes clinically feasible for T1D treatment.

## 1. Introduction

Type 1 diabetes (T1D) is an autoimmune disease that results from T cell-mediated destruction of pancreatic beta cells. Autoantibodies produced by autoantigen-specific B lymphocytes can be detected in individuals with T1D years prior to clinical diagnosis [1]. However, in contrast to other autoimmune diseases, such as systemic lupus erythematosus, autoantibodies are not considered to be inherently pathogenic in T1D [2,3]. Instead, B cells function as critical antigen presenting cells (APCs), and drive T1D by presenting islet-derived autoantigens to T cells [3,4,5,6]. Systemic immunosuppression can subdue these destructive T-B cell interactions and induce T1D remission or slow disease progression [7,8,9,10]. However, T1D is most commonly diagnosed in children [11,12] who may have yet to be fully vaccinated at diagnosis; therefore, targeting diabetogenic immune interactions while sparing protective immune responses is particularly important. One therapeutic approach to achieve this goal is to disrupt autoantigen-specific T-B cell cross talk to prevent autoimmune destruction in T1D. Here, we will review the current status of antigen-specific B cell-targeted therapies for T1D and expound on what may be required for the development of successful therapeutic intervention.

## 2. T-B Lymphocyte Interactions Promote T1D

In 1986, George Eisenbarth proposed that T1D was a chronic autoimmune disease caused by an unknown environmental trigger in genetically susceptible individuals and resulted in progressive loss of beta cell function. In this seminal work, he observed that immune targeting of several islet autoantigens preceded clinical diagnosis of T1D [13]. Currently, T1D research consortium TrialNet screens first-degree relatives of individuals with T1D for islet autoantibodies against insulin, GAD65, IA-2, ICA512, and ZNT8 autoantigens; positivity for two or more of these specificities confers an ~80% risk of developing diabetes within 20 years [1]. Islet-specific autoantibodies herald disease onset; however, they are not pathogenic [14]. Because beta cell destruction has classically been considered T cell mediated, many interventions have targeted the T cell response. Recent examples tested in T1D clinical trials include the T cell depleting anti-CD3 monoclonal antibody, teplizumab, and the costimulatory modulating CTLA-4/Fc fusion protein, abatacept [10,15].

A role for B lymphocytes in propagating autoimmune beta cell destruction by T cells has also been demonstrated. B lymphocytes provoke islet attack in mice by presenting autoantigens to activate cognate T cells [3,4,5,6,16,17]. Neutralization of BAFF, a B lymphocyte survival factor, prevented T1D in non-obese diabetic (NOD) mice [18], as did total B lymphocyte elimination [19]. In humans, increased B lymphocyte infiltration of islets corresponds with more aggressive and earlier disease onset [20]. B lymphocyte depletion through a short course of an anti-CD20 monoclonal antibody temporarily preserved beta cell function in individuals with recent T1D onset, solidifying a role for B lymphocytes in human T1D [21]. Orchestration of a successful autoimmune attack requires T-B cell cross-talk, and disruption of this cross talk between antigen-specific B and T lymphocytes remains an unrealized therapeutic goal.

## 3. Therapies That Broadly Target T-B Lymphocyte Interactions

Global elimination of T or B lymphocytes has seen transient clinical success, in part due to the limited period of intervention. Modulation of T cell co-stimulation with abatacept was shown to reduce the decline in beta cell function over two years in individuals with recent onset T1D [15], and the recent, encouraging, results of the teplizumab study, in which treating individuals positive for islet-specific autoantibodies but prior to clinical disease with the anti-CD3 monoclonal antibody teplizumab delayed disease onset by two years [10]. In 2009, Pescovitz and colleagues reported the results of the first phase 2 study to evaluate the role of B cell depletion using the anti-CD20 monoclonal antibody, rituximab, in new-onset T1D patients. At 12 months, individuals who received the four-dose course of rituximab (which occurred during the first 22 days of the trial) were found to have partially preserved beta cell function as evidenced by decreased C-peptide loss, reduced insulin requirements, and lower HbA1c [21]. However, follow-up evaluation at 24 months revealed no significant difference in C-peptide loss between those treated with rituximab compared to placebo [22]. Autoreactive and polyreactive B lymphocytes remained elevated in T1D patients before and after treatment, and rituximab failed to prevent the re-emergence of newly generated B cell clones or alter early B cell tolerance checkpoints [23]. Rituximab treatment did however suppress insulin autoantibodies (IAAs), but not GAD, IA-2, or ZnT8 autoantibodies; sub-analysis revealed that lower IAAs tracked with “responders” that were defined based on C-peptide levels during follow-up, independent of treatment regimen [24]. It is possible that multiple courses of rituximab to sustain B cell depletion could have changed these outcomes; however, as is the case for any broadly depleting cellular therapy, its use is limited by the risk of side effects associated with sustained immune suppression. Notably, protective vaccine responses were suppressed in rituximab-treated individuals, questioning the safety of this approach in a pediatric population [25].

In addition to inherent risk associated with immune suppression, global lymphocyte depletion may also alter downstream immune responses to impede efficacy. To further delineate the transient nature of global B cell depletion observed in rituximab trials, Serreze and colleagues used human and murine anti-CD20 as a B lymphocyte depleting agent in NOD mouse models to demonstrate that B lymphocytes downregulate CD20 upon entry into the islet, which impedes the capacity for anti-CD20 treatment to affect disease at later stages [26]. Conversely, work by Boldison et al. suggests that downregulation of CD20 is not universal for all islet-infiltrating B cells. Instead, autoreactive, AIBCs comprise a heterogeneous population in which CD20^+^ AIBCs preferentially home to islets in the pancreas where they adopt a distinct CD138^int^ phenotype [27]. After global B lymphocyte depletion, these antigen-specific B lymphocytes repopulate the islets sooner than non-antigen-specific B lymphocytes, which supports the notion that antigen-specific, targeted therapy will be required for improved efficacy and safer therapeutic alternatives [27].

## 4. Exogenous Insulin Provision to Alter Immune Recognition and Prevent T1D

Insulin is a major autoantigen in T1D: IAAs signal that pathogenic T-B interactions have occurred and predict T1D in both mice and humans [28,29,30]. Insulin recognition by both T and B lymphocytes is required in T1D-prone NOD mice [16,31,32]. It is not surprising, then, that many of the strategies proposed to induce tolerance in individuals with or at risk for T1D use insulin or insulin peptides. Insulin therapy has effectively prevented diabetes development in several animal models by suspected metabolic and immunologic mechanisms [33,34,35]. The Diabetes Prevention Trial-Type 1 (DPT-1) was the first randomized, multicenter trial that asked whether T1D could be prevented or delayed in high risk individuals by insulin administration prior to disease onset [36,37]. Over 80,000 first- and second-degree relatives of patients with T1D were screened. Those with a projected five-year risk of T1D development were randomly assigned to either daily low dose subcutaneous insulin and annual IV insulin infusions, or to close observation. No differences in progression to diabetes were observed between the two groups [37]. Follow-up analysis demonstrated that IV insulin did indeed provide the hypothesized “beta cell rest” by suppressing endogenous insulin secretion; however, the subcutaneous insulin regimen did not [36]. Previous work demonstrated that, in addition to suppression of endogenous insulin secretion, parenteral insulin induced suppression of T cell proliferation to islet antigens; however, the effect was transient [38].

Because antigens encountered via the enteral route typically elicit immune tolerance, the effects of intranasal and oral insulin on T1D progression have been studied in multiple cohorts of patients with, and at high risk for, T1D. A pilot study of 38 prediabetic, autoantibody-positive individuals treated with intranasal insulin, published in 2004, found that intranasal insulin increased IAAs, decreased T cell proliferation, and, importantly, did not accelerate beta cell loss [39]. In 2005, Skyler and colleagues published the results of a randomized, double-masked, placebo-controlled trial to test whether oral insulin could prevent or delay T1D in high risk relatives [40]. First or second degree relatives who were ICA+ and IAA+ and with a first phase insulin response above the threshold (≥100 μU/mL if age ≥8 years; ≥60 μU/mL if age <8 years for siblings, offspring, and second-degree relatives; ≥60 μU/mL for parents) were enrolled into the DPT-1 trial to the test preventative effects of 7.5 mg/d oral insulin [40]. A 5-year delay of diabetes onset was observed in a subgroup that had the highest IAA titer [40]. A trial to replicate the findings in this subset focused on relatives who were positive for at least two islet autoantibodies (including IAA) [41]. No significant delay in diabetes onset was observed overall between 7.5 mg/d oral insulin and placebo groups [41]. However, subgroup analysis revealed a significant, nearly three-year delay in diabetes onset in the group in which first-phase insulin response was lower than the threshold.

The Pre-POINT study, which looked at genetically at-risk children without evidence of autoimmunity and used immune responses as outcome measures, only observed protective immunomodulatory effects in children who received the highest oral insulin dose of 67.5 mg/d [42]. The primary outcome for this study was a positive immune response to insulin, including an increase in serum IgG IAAs, salivary IgA IAAs, or a positive CD4+ T cell response to insulin in peripheral blood. This was the first oral insulin trial to characterize the immune responses in treated children using composite analysis of B and T cell responses to insulin, and the first to document the presence of IAAs in the saliva, suggesting that the immune response originated from the oral mucosa. In insulin-treated children, insulin and proinsulin responsive regulatory T cells were induced, and IL-21 expressing insulin (but not proinsulin) positive CD4+ T cells were identified, suggesting that treatment with oral insulin may promote T-B lymphocyte interactions that enhance regulatory T cell development [42].

## 5. AIBCs Drive T1D

**Figure 1:**
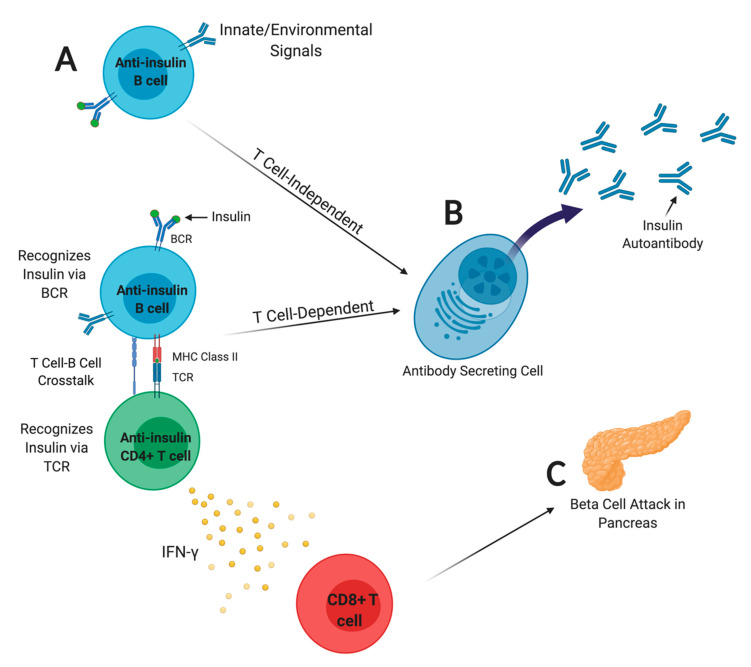
T-B lymphocyte crosstalk precedes autoimmune attack of pancreatic beta cells and insulin autoantibody (IAA) production. (**A**) Anti-insulin B cells (AIBCs) present insulin autoantigen to anti-insulin T cells to drive their expansion [45,46]; (**B**) IAA detected in peripheral blood is a major biomarker used to predict and stratify T1D risk [1,53,54]. AIBCs can differentiate into IAA-secreting cells if they receive the right signals to promote immune checkpoint breach. T-independent but not T-dependent immunization drives AIBCs to differentiate into IAA-secreting cells in VH125^SD^/NOD mice, which secrete little IAA spontaneously [46,49]. Provision of a large pool of anti-insulin T cells via the 8F10 T cell receptor (TCR) transgene drives VH125^SD^ AIBCs to produce IAAs [50]; thus, T-dependent stimulation of IAA production is possible. (**C**) CD4^+^ T cells secrete IFN-γ, which in turn promotes CD8+ T cell acquisition of effector function and beta cell attack. Original image, created with BioRender.com.

While T cells are the primary mediators of beta cell destruction, their activation and expansion are the result of antigen presentation by professional APCs, including dendritic cells, macrophages, and B lymphocytes. The ability of B lymphocytes to internalize and process small concentrations of specific antigen and present it via peptide-MHC class II complexes to autoreactive T cells is unique among professional APCs [43]. B lymphocyte-specific deletion of MHC class II prevents T1D in the NOD mouse model [3]. Furthermore, B lymphocyte specificity is a powerful driver of disease onset in the NOD mouse. B lymphocyte repertoires biased toward insulin accelerate disease onset while those biased toward an irrelevant antigen are protected [16]. Anti-insulin T cells adopt a default regulatory phenotype but become pathogenic in the presence of an expanded pool of insulin specific B cells in mice, and there is a direct correlation between insulin specific effector memory T cells and IAA titers in individuals with new onset T1D [27,44]. It is not surprising, then, that anti-insulin B cell receptor (BCR) heavy chain transgenic mice (VH125^SD^/NOD) develop accelerated insulitis and diabetes secondary to an increased frequency of AIBCs, whereas NOD mice lacking AIBCs are protected [16,45,46]. VH125^SD^/NOD mice have a larger percentage of AIBCs (~1–2%) in secondary lymphoid organs relative to WTNOD mice, in which they are frequently undetectable [46,47]. Interestingly, VH125^SD^/NOD mice produce little IAA, either spontaneously or following T-dependent immunization, yet can upregulate co-stimulatory molecules such as CD86 and proliferate to T-independent and T-dependent stimuli [46]. The incomplete anergy observed in AIBCs contrasts other autoreactive B cells in which developmental and functional blocks are more severe [48]. Thus, AIBCs can drive T1D through their function as APCs, despite being functionally silenced for IAA production [45,46]. In contrast to T-dependent immunization, T-independent immunization with Brucella-conjugated insulin drives AIBCs to escape immune checkpoints to differentiate into IAA-secreting cells in VH125^SD^/C57BL/6 mice [49]. Whereas insulin does not drive AIBC proliferation in this model, LPS (but not anti-CD40) synergizes with insulin stimulation through the BCR to drive proliferation [49]. Anti-insulin (8F10) T cells can drive anti-insulin (VH125^SD^) B cells to secrete class-switched IAAs, thus T-dependent IAA production can occur [50]. The routes by which AIBCs expand, differentiate into IAA-secreting cells, and drive T cell-mediated beta cell attack are outlined in Figure 1.

In humans, AIBCs are absent from the anergic (B_ND_) subset in at-risk and new onset-T1D individuals [51]. Smith et al. postulated an environmental event may precipitate T1D, as all B_ND_ B lymphocytes transiently disappear around the time of T1D onset [51]. This loss of anergy is associated with both high-risk HLA alleles and non-HLA risk alleles involved in B and T lymphocyte development which supports a role for genetic predisposition in B lymphocyte tolerance breaches in T1D [52]. Together, these studies demonstrate the threat that AIBCs pose to disease development in both mouse and human T1D.

## 6. T-B Lymphocyte Interactions in T1D Differ from Classic Protective Immunity

High-affinity IAAs are found in people at risk for T1D [55]. It is unclear whether such affinity maturation of B cell responses is required for T1D development, or whether it is a natural consequence of long-standing autoimmune responses that may have raged for years in the pre-symptomatic disease interval. Germinal center B cells are uniquely able to invoke cognate anti-insulin T cell proliferation due to differences in insulin processing and presentation, suggesting that germinal center B cells may be instrumental in provoking immune tolerance breach by T cells [50]. Evidence is mounting, however, that suggests minimally-matured responses are capable of driving T1D pathogenesis. Activation-induced cytidine deaminase (AID) is required for class-switching and somatic hypermutation of BCRs [56]. Conflicting studies report diabetes exacerbation [57] or protection [58] in AID-deficient NOD mice. AID also promotes central tolerance [59], a role which is difficult to dissect from its function later in B cell maturation/activation. AIBCs that cannot undergo class switch recombination still support T1D development [16,60]; class switch recombination is not required for their function as APCs [45].

mAb125, from which the anti-insulin 125Tg BCR is derived, harbors two CDR2 mutations in VH125 that confer modest rodent insulin recognition (8 × 10^6^ M^−1^), yet 125Tg B cells support diabetes in NOD mice [60,61]. BCRs can interact with antigen bivalently, thus both affinity and avidity can impact the strength of BCR signaling. Differences in avidity for insulin can overcome low-affinity interactions of the antibody when studied in solution; as such, the 125Tg BCR, which has low affinity but high avidity for insulin, shows higher BCR occupancy in the presence of the same insulin concentration in vitro compared to the higher affinity but lower avidity A12 BCR that pairs VH125 with a different light chain [62]. Repetitive antigen epitopes enhance BCR cross-linking to augment BCR signaling. Insulin can dimerize or hexamerize (e.g., in beta cell secretory granules) but exists as a monomer at physiologic concentrations [63,64]. It is unclear whether insulin bound to AIBC BCRs achieves an effective local concentration that permits dimerization, which depends on accessibility of the insulin F-F-Y “aromatic triplet” [65]. Autoreactive B cells can also exhibit signaling differences, reviewed in [66]. Thus, many biologic parameters, including BCR/antigen binding strength, antigen valency, BCR downregulation, and intrinsic B cell signaling differences can affect binding interactions and cell signaling responses dynamically.

SLAM-associated protein (SAP) is necessary for classic germinal center reactions and affinity-matured antibody responses [67,68,69,70]. Germinal center B cells are reduced in SAP-deficient NOD mice, yet organized tertiary lymphoid structures form in the islets unabated, and T1D still develops in ~50% of mice [71]. Numerous islet-specific T cell receptors (TCRs) isolated from NOD mice exhibit weak binding to cognate peptides [72,73,74]. Taken together, these data suggest B and T cells with low or moderate affinity for autoantigen may engage each other productively to initially drive T1D autoimmunity. Affinity-matured B lymphocyte responses that stem from T follicular help in germinal centers may arise as a natural byproduct of islet attack driven via these initial primary responses.

## 7. Therapeutic Targeting of AIBCs

**Figure 2:**
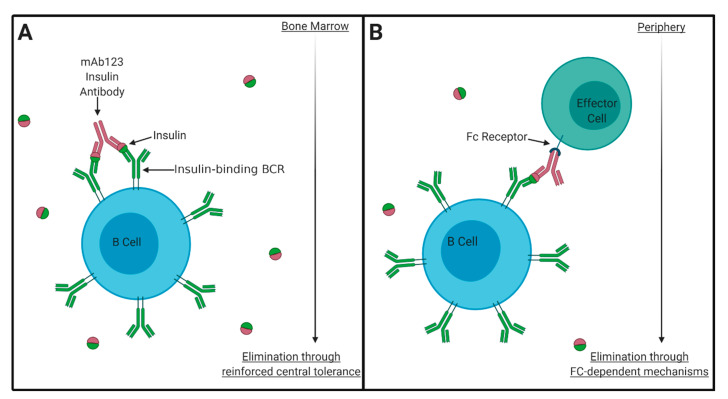
Fighting fire with fire: using anti-insulin mAbs to target AIBCs. Insulin-occupied BCRs are found on the surface of mouse B cells that express transgenic anti-insulin BCRs. This can be exploited by using an anti-insulin antibody (mAb123) that binds a separate epitope of the BCR to selectively eliminate AIBCs from the repertoire and prevent T1D in NOD mice [47]. Two mechanisms have been shown to drive this depletion. (**A**) Enhanced BCR crosslinking reinforces central tolerance in the bone marrow. This Fc-independent function improves receptor editing to limit AIBC escape into the periphery [81]. (**B**) Fc recognition provokes AIBC elimination, presumably via antibody-dependent cell-mediated cytotoxicity, a mechanism of action for other antibody-based biologic therapies. Original image, created with BioRender.com.

Elimination of autoantigen-specific B cells is an ideal therapeutic alternative to global B cell suppression or depletion. In children, the appearance and levels of IAA specifically correlate with T1D development [28]. Therefore, elimination of AIBCs has the potential to impact early T-B cell interactions. Monoclonal antibodies (mAbs) specific for different insulin epitopes permit the detection of insulin-occupied BCRs, and, therefore, AIBCs [75]. Murine mAb123 and mAb125 are IAAs that can simultaneously bind to separate insulin epitopes [76]. As such, mAb123 detects insulin bound to the 125Tg and VH125Tg BCRs [75,77]. Treatment with anti-insulin mAb123 selectively eliminates AIBCs but preserves the broad B cell repertoire [47]. T1D is prevented in NOD mice treated with mAb123 every other week to prevent repopulation by AIBCs that continuously emerge from the bone marrow [47,77]. Interestingly, whereas AIBCs present in the spleen and lymph nodes are detected through staining with labeled insulin, AIBCs in the pancreas are not [47,78]. Labeled mAb123 detects AIBCs in the pancreas, showing insulin occupancy of AIBC BCRs in the pancreas in increased, thus limiting their detection by labeled insulin, but likely enhancing their targeting by mAb123 [77]. Treatment with mAb123 more effectively eliminated mature AIBCs compared to immature AIBCs, despite their enhanced sensitivity to BCR signaling [79]. This is presumably due to enhanced Fc-mediated elimination in the periphery. Rituximab eliminates B cells via antibody-dependent cell-mediated cytotoxicity [80]. The autoantigen-targeting approach of mAb123 holds additional functionality beyond Fc recognition: F(ab’)2 123 (which lacks the Fc domain) reinforces central tolerance in the bone marrow via receptor editing to enhance clearance of AIBCs from the repertoire, presumably through enhanced cross-linking of BCRs bound monovalently to insulin [81] (Figure 2). Soluble antigen arrays, in which insulin is conjugated to polymers, have also been used to reinforce immune tolerance in AIBCs [82].

Alteration of BCR signaling also shows therapeutic promise in NOD mouse models. Bruton’s tyrosine kinase (BTK) is an important mediator of BCR signaling and therefore B cell fate and function [83]. In NOD mice, *Btk* deficiency protects against diabetes and prohibits IAA production without significant reduction in B cell number [84]. Total serum IgG is unchanged by *Btk* deficiency, suggesting a selective effect on autoreactive, AIBC function. This protection is reversed by introduction of an anti-insulin BCR H chain transgene (VH125), indicating that *Btk* deficiency protects against T1D by promoting a reduction in autoreactive specificities within the B cell repertoire. Autoreactive AIBCs rely more heavily on BTK than non-autoreactive B cells and are preferentially affected by *Btk* deficiency [85]. Numbers of autoreactive B cells are reduced in the naturally occurring polyclonal repertoire of An1 B cells in *Btk*-deficient mice. In anti-insulin BCR transgenic NOD mice (125Tg/NOD), whose B cell repertoire is comprised nearly universally of AIBCs, B cells in the spleen were reduced by 95%. The decrease is more pronounced in mature AIBCs. In an Igκ locus site-directed model, where 50% of BCRs bind insulin and 50% are edited to non-insulin specificities, AIBCs are selectively reduced in the follicular and marginal zone B cell subsets, revealing preferential dependence of AIBCs on BTK [85]. *Btk* deficiency, however, does not appear to affect their function, and the few remaining mature AIBCs are able to internalize antigen, traffic to the pancreatic lymph nodes and spleen, and cause T1D in 125Tg/NOD mice [85]. Inducible deletion of *Btk* impacts AIBC development, but not survival [86]. Consequently, BTK targeting in NOD mice is unlikely to impair autoreactive, AIBC function but instead decreases the availability of mature, autoreactive, antigen-specific B cells within an endogenous repertoire. As mentioned above, AIBCs repopulate the pancreas earlier than non-insulin-binding B cells following anti-CD20 B cell depletion [27]. This suggests that an ideal therapeutic strategy may require a large hit to B cells (e.g., via rituximab) followed by maintenance therapy via BTK inhibition to prevent re-emergence of AIBCs.

The NFAT transcription factor family provides another example of how targeting AIBC signaling has the potential to promote tolerance. This was demonstrated by introducing a functionally inactive *NFATc2* into C57BL/6 mice transgenic for an anti-insulin BCR (125Tg) [87]. The B cell repertoire in wild type 125Tg mice is composed of anergic AIBCs. Without NFATc2, however, AIBCs lose their anergic phenotype in response to BCR stimulation. B cell maturation, surface BCR expression, and IAA production were not changed by the functional absence of NFATc2. Increases in NFATc1 and NFATc3 were observed with the release of anergy in AIBCs, indicating that NFAT transcription factors play specific and selective roles in maintaining B cell tolerance [87]. Understanding the signaling mechanisms that drive tolerance in antigen-specific B cells is critical to expanding the opportunities for selective elimination of pathogenic B cell specificities in T1D and other autoimmune diseases.

Developing AIBCs in the bone marrow are subject to anergy and receptor editing, and peripheral tolerance is maintained via an incomplete form of anergy, albeit insufficiently to prevent diabetes [60,75,81,88]. Immunoglobulin polymorphisms enhance insulin recognition in NOD mice, which is complicated further by less efficient receptor editing of AIBCs compared to an autoimmune strain [77,81]. Defective immune tolerance in NOD mice is further highlighted by the increased frequency of AIBCs that reach the periphery of NOD mice, compared to C57BL/6 mice that express the same BCR transgene and the NOD MHC class II allele, IAg7 [89]. NOD transitional B cells that express the same BCR show less BCR downregulation following culture with insulin compared to those isolated from non-autoimmune C57BL/6 mice [62]. Lower affinity AIBCs that undergo less IgM downregulation following co-culture with insulin elicit a higher frequency of T cells to secrete the inflammatory cytokine IFN-γ in vitro [62].

## 8. Targeting Autoreactive B Cell Developmental Subsets

While insulin has been identified as a critical autoantigen, the list of diabetes-associated autoantigens continues to grow and now includes post-translationally modified neoepitopes and hybrid insulin peptides, generated at the site of autoimmune attack in the islet [90]. This amplification of the antigenic repertoire challenges the concept of antigen-specific therapy with a single autoantigen. As such, removal of autoreactive cells, regardless of antigen-specificity, may be an alternative approach that provides more selective targeting than global immune suppression. Using anti-insulin BCR transgenic VH125^SD^/NOD mice, our group showed that, in a polyclonal repertoire, AIBCs are preferentially skewed into marginal zone and late transitional subsets known to have increased sensitivity to proinflammatory signals [46]. Furthermore, the developmental fates of AIBCs were different for high affinity compared to low affinity insulin-binding. Our results demonstrated the capacity of AIBCs to enter all mature B cell subsets, but show that they are enriched in the marginal zone subsets compared to non-autoreactive, non-insulin binding cells. AIBCs with higher insulin affinity were found to be enriched in T2 subsets in the spleen and PLNs. Only lower-affinity AIBCs developed into follicular subsets, indicating that BCR affinity for self-antigen influences AIBC developmental fate [46]. The transitional B cell stage is an important peripheral tolerance checkpoint that has been shown to be relaxed in NOD mice, leading to the failure of elimination of autoreactive B cells at this developmental stage [91,92]. However, transitional B cells have also been shown to have immunoregulatory roles, capable of suppressing IFN-γ and CD4+ T cell proliferation in an IL-10 dependent manner in other models of autoimmune disease [93]. In VH125^SD^/NOD mice, IL-10 production is increased in AIBCs [46]. Therefore, in addition to targeting autoreactive B cell subsets for elimination, expansion of regulatory B cell subsets provides an additional therapeutic target.

Differences in peripheral B cell subsets are not limited to mouse models of T1D. Immunophenotyping of circulating B cells was used to demonstrate that some T1D individuals had reduced proportions of B cells expressing TACI and Fas maturation markers and B cell subsets with lower frequencies of class switching compared to healthy donors, suggesting differential B cell selection in T1D [94]. Transitional, naïve, activated, and memory cell subsets were not different between T1D and healthy donors, thus egregious differences were not apparent between T1D and healthy controls [94]. In children with T1D, the percentages of circulating IL-10 producing and immature transitional subsets of regulatory B cells were lower than in healthy controls [95]. Furthermore, genetic analysis of B cell subsets in individuals with T1D compared to healthy controls identified an association between IL-10 production by B cells and the diabetes risk allele IL2-IL21. Individuals with this risk allele had decreased production of IL-10 by both memory B cells and by autoreactive CD4+ T cells [96].

## 9. Challenges to B Cell Directed Therapy

Autoantibodies are powerful predictors of future disease in individuals at risk for T1D, and, as a result, nearly universally, intervention trials recruit individuals who are already autoantibody positive. B lymphocytes must receive the right stimulation to differentiate into plasmablasts or plasma cells that secrete BCR as a circulating antibody. Different immune checkpoints govern whether autoreactive B cells (1) expand, (2) undergo mutation and affinity maturation, and (3) differentiate into antibody-secreting cells [97]. Thus, autoreactive B cells can retain disease-relevant antigen-presenting function in mice even when their differentiation into antibody-secreting cells is blocked [45,46,60]. Furthermore, autoantibody production indicates pathogenic T-B cell interactions have already occurred and that pathogenic, autoreactive T cell clones have been expanded. Dampening the autoimmune response, even before clinical disease is diagnosed, remains a difficult hurdle to clear. The development of novel immunologic and metabolic biomarkers will undoubtedly aid in earlier identification of at-risk participants, hasten our ability to discern favorable responses to novel therapies, and better identify optimal timing of interventions. Increasingly appreciated disease heterogeneity associated with T1D progression and development further complicate not only understanding disease pathogenesis, but also identifying individuals who should be enrolled in clinical trials. Detailed studies of early immunologic underpinnings of T1D are essential to unlock the mechanisms behind this heterogeneity in the future.

## 10. Conclusions

While T1D is classically considered a T cell-mediated disease, B cells are indispensable for disease onset in NOD mice and play an imperative yet incompletely understood role in human T1D. Here, we have reviewed the unique features and function of antigen-specific B cells and their role in T1D progression. The success of broad T and B cell depletion therapies to date demonstrates that immune intervention has encouraging potential. We propose that targeting pathogenic B cell specificities for elimination, or specific regulatory B cell subsets for expansion, has the potential to improve both the safety and efficacy of immunotherapy in T1D.

## Data Availability

Not applicable.

## Abbreviations

antigen presenting cell (APC), anti-insulin B cell (AIBC), B cell receptor (BCR), Bruton’s tyrosine kinase (BTK), insulin autoantibody (IAA), monoclonal antibody (mAb), non-obese diabetic (NOD), SLAM-associated protein (SAP), T cell receptor (TCR), and type 1 diabetes (T1D).
